# The involvement of NETs in ANCA-associated vasculitis

**DOI:** 10.3389/fimmu.2023.1261151

**Published:** 2023-09-14

**Authors:** Satoka Shiratori-Aso, Daigo Nakazawa

**Affiliations:** Department of Rheumatology, Endocrinology, and Nephrology, Faculty of Medicine and Graduate School of Medicine, Hokkaido University, Sapporo, Japan

**Keywords:** ANCA-associated vasculitis, neutrophil extracellular traps, necrosis, reactive oxygen species, treatment

## Abstract

Anti-neutrophil cytoplasmic autoantibody (ANCA)-associated vasculitis (AAV) is a serious autoimmune disease that is characterized by vascular necrosis. The pathogenesis of AAV includes ANCA-mediated neutrophil activation, subsequent release of inflammatory cytokines and reactive oxygen species (ROS), and formation of neutrophil extracellular traps (NETs). Excessive NETs could participate not only in ANCA-mediated vascular injury but also in the production of ANCAs per se as autoantigens. Thus, a vicious cycle of NET formation and ANCA production is critical for AAV pathogenesis. Elucidating the molecular signaling pathways in aberrant neutrophil activation and NETs clearance systems will allow specific therapeutics to regulate these pathways. Currently, standard therapy with high doses of glucocorticoids and immunosuppressants has improved outcomes in patients with AAV. However, AAV frequently develops in elderly people, and adverse effects such as severe infections in the standard regimens might contribute to the mortality. Mechanistically, cytokines or complement factors activate and prime neutrophils for ANCA-binding; thus, C5a receptor blocker has garnered attention as potential replacement for glucocorticoids in clinical settings. Recent studies have demonstrated that receptor-interacting protein kinases (RIPK3) and cyclophilin D (CypD), which regulate cell necrosis, may be involved in ANCA-induced NETs formation. Meanwhile, targeting NETs clearance, including the addition of deoxyribonuclease I (DNase I) and macrophage engulfment, may improve vasculitis. In this review, we focus on the pathogenesis of NETs and discuss potential targeted therapies for AAV based on recent experimental evidence.

## 1 Introduction

Anti-neutrophil cytoplasmic antibody (ANCA)-associated vasculitis (AAV) is an autoimmune disease characterized by necrotizing inflammation of small blood vessels. Crescentic glomerulonephritis and pulmonary hemorrhage are serious complications of AAV. AAV is subdivided into microscopic polyangiitis (MPA), granulomatosis with polyangiitis (GPA), and eosinophilic granulomatosis with polyangiitis (EGPA) (1). Proteinase 3 (PR3)-ANCA and GPA are more common in western countries, whereas myeloperoxidase (MPO)-ANCA and MPA are more common in East Asian countries (2). Loss of tolerance to MPO or PR3 leads to the generation of ANCAs (1), and humoral factors including ANCAs activates neutrophils and form neutrophil extracellular traps (NETs) (2). Incubation of tumor necrosis factor (TNF)-α-primed human neutrophils with ANCA-IgG from AAV patients leads NET formation (3, 4). Similarly, murine peripheral neutrophils form NETs by stimulation with TNF-α and anti-MPO antibody (4, 5). NETs consist of extracellular deoxyribonucleic acid (DNA) fibers comprising histones and antimicrobial proteins such as MPO and PR3 as autoantigens of ANCAs (6). Histones released by NETs induce endothelial cell injury (7). Currently, standard therapy with glucocorticoids and immunosuppressants has improved outcomes in AAV. However, the adverse effects of treatment contribute to mortality and morbidity (8); in particular, AAV requires a pathogenesis based approach to replace steroid. Considering the development of AAV, NETs are potential therapeutic targets. Herein, we summarize and discuss the current and future potential targeted therapies against NETs in AAV by referring to recent experimental studies.

## 2 Overview of NETs and NETosis

NETs are essential elements of the host innate immune response against microbial infections and they effectively capture and kill invading pathogens. NETs are net-like structures composed of DNA fibers, histones, and antimicrobial proteins such as MPO and neutrophils elastase (NE) which are released from activated neutrophils (6). NET formation is accompanied by a unique form of neutrophils cell death called NETosis. NETs are formed *via* various signaling pathway in response to different triggers (9). Stimuli such as microbes, immune complexes (ICs), certain autoantibodies, and cytokines activate nicotinamide adenine dinucleotide phosphate (NADPH) oxidase (NOX) *via* immunoreceptors and produce ROS, and are involved in suicidal NETosis. Phorbol myristate acetate (PMA), an activator of protein kinase C (PKC), induces NOX-dependent suicidal NETs *via* Raf-MEK-ERK pathway (10). ROS initiates the translocation of NE and MPO from granule to nuclei and participate in the rupture of nuclear membrane. Furthermore, ROS activate peptidylarginine deiminase 4 (PAD4) (11, 12), which converts histone arginine to citrulline, resulting in chromatin decondensation in the nuclei of neutrophils (13, 14). The nuclear and granular membranes disintegrate, and then the decondensed chromatin mixes freely with the contents of the granules and cytoplasmic materials. Finally, this decondensed chromatin is finally expelled outside neutrophils accompanied by cellular lysis (15).

In contrast, some microorganisms and activated platelets induce NETs without cell lysis, termed vital NETosis. Vital NETosis occurs faster than suicidal NETs, and its pathways is assumed to be NOX-independent (16). Neutrophils stimulated with granulocyte/macrophage colony-stimulating factor (GM-CSF), lipopolysaccharide (LPS) and C5a form vital NETs with release of mitochondrial DNA (17).

Recent studies have revealed that neutrophils are a heterogenous population with distinct behaviors and are involved in the disease including autoimmune diseases (18, 19). Low-density granulocytes (LDGs) are a heterogeneous population of mature and immature neutrophils (20). LDGs synthesize increased levels of proinflammatory cytokines, and are prone to spontaneously form NETs compared with normal-density granulocytes (NDGs) (21). However, LDGs in AAV are hyporesponsive to anti-MPO antibody stimulation (22). In most studies, ex vivo experiments on NETs induction have been conducted using bulk NDGs; therefore further studies are needed to elucidate the role of NETs on the basis of cell subsets.

## 3 The role and mechanism of NETs in AAV

AAV is a systemic autoimmune disease characterized by the presence of ANCAs, which are pathogenic autoantibodies against MPO, PR3, or other neutrophil-derived molecules. NETs have been shown to be present in the kidney (3), nervous system (23), and pulmonary capillaries (24) in patients with AAV. Neutrophils are primed by inflammatory mediators including TNF-α, interleukin (IL)-1β, and complement C5a. Primed neutrophils express ANCA antigens including MPO or PR3 on their cell surface. The Fab and Fc regions of ANCAs bind to their antigens and Fcγ receptors, respectively, on neutrophils. Based on several studies, intracellular signaling after ANCA binding is supposed to be as follows: i) The Fc region crosslinks with Fcγ receptor coupled with integrin, resulting in the activation of spleen tyrosine kinase (Syk) and generation of NOX-mediated ROS (25). ii) ROS initiates the activation of PAD4, which mediates chromatin decondensation, iii) NE and MPO breaks nuclear and cellular membranes, finally leading to the release of NETs. During these processes, receptor interacting protein kinases (RIPK3)/mixed linage kinase domain-like protein (MLKL) (necroptosis regulator) and cyclophilin D (CypD) (mitochondria-driven necrosis regulator) might be involved in the rupture of membrane as a necrosis conductor (5, 26). Moreover, the cellular adhesion to endothelium *via* integrin and subsequent cytoskeletal alteration is required for ANCA-induced NETs formation (27). These ANCA-mediated NETs with cytotoxic properties initiate endothelial injury in coordination with complement systems, coagulant system, and other immune cells (4, 7). Some studies have shown that neutrophils treated with serum derived from AAV patients produce high amounts of NETs with histone citrullination. However, these NETs are not affected by depletion of IgG/IgA or blocking of C5 activation (28–30); thus, the mechanisms of humoral factors in NET formation are not fully understood (31).

Meanwhile, under steady state, soluble NETs-DNA and netting neutrophils are processed by serum deoxyribonuclease (DNase) I (32) and phagocytic cells, respectively (33, 34). However, patients with AAV have low activity of serum DNase I and netting neutrophils with phagocytosis-resistance (35, 36), implying that unprocessed NETs might persistent in body and serves as autoantigens against ANCA, which contributes to the development of disease. In support of this hypothesis, propylthiouracil (PTU), a medication causing drug-induced AAV, induces DNase I-resistant NETs with the modification of MPO structure in ex vivo (37) and the administration of these PTU-treated NETs promotes the generation of MPO-ANCA *in vivo* (38). Meanwhile, evidence of human data suggests that the increased expression of PR3 and MPO associated with epigenetic alteration contributes to the development of AAV (39, 40). Furthermore, several human leukocytes antigen (HLA) susceptibility alleles associated with AAV have been identified by genome-wide association studies (GWAS) (41) and individuals with these risk alleles might recognize extracellular MPO and PR3 as autoantigens. This speculation is corroborated by the case series demonstrating that MPO-AAV developed in individuals with risk allele after the administration of drug or COVID-19 vaccine (42, 43). Taken together, the disease onset, particularly ANCA development, might be due to a combination of factors including dysregulation of NETs, epigenetic alterations of antigens, and genetic susceptibility to disease.

## 4 Targeting NETs signaling pathway in AAV

**Figure 1:**
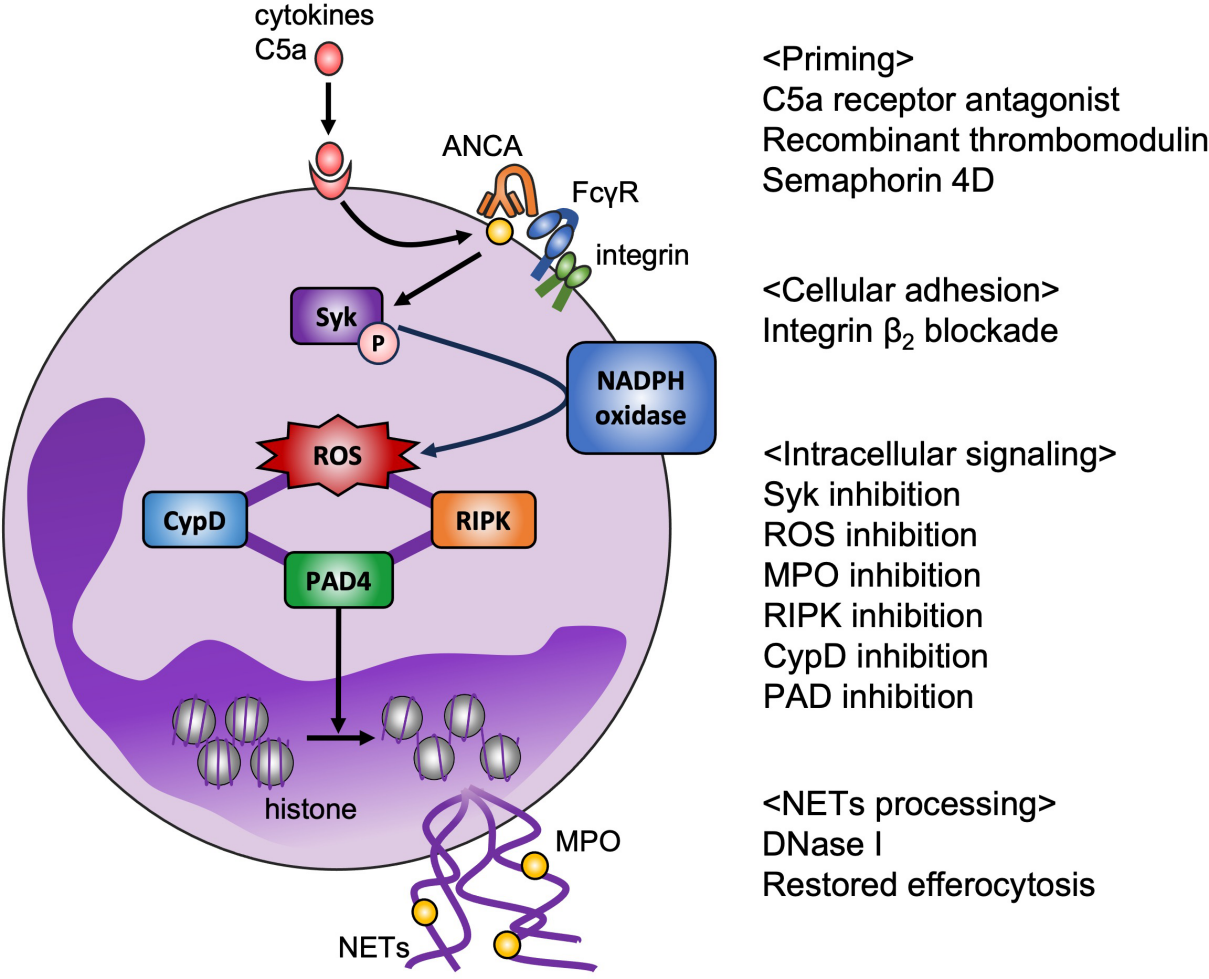
Potential therapeutic targets for AAV based on *ex vivo* model. NETs play a key role in pathogenesis of AAV. Disease-specific strategies for targeting NETs are required to reduce adverse effects. Binding C5a to its receptor on neutrophils translocates MPO to the cell surface, which is a target of avacopan (C5a receptor antagonist). Recombinant thrombomodulin binds to integrin β2 on neutrophils, and semaphorin 4D on neutrophils binds to plexin B2 on endothelial cells, resulting in suppression of neutrophil priming. Integrin-mediated cell adhesion is also essential for NET formation *in vitro*. Syk phosphorylation promotes production of NOX-mediated ROS, which are the key factor in NET formation, and inhibition of MPO decreases ROS production. RIPK and CypD are assumed to be involved in the rupture of membrane. PAD4-mediated histone citrullination induces chromatin decondensation. Inhibition of these intracellular signaling could prevent NET release. From the perspective of NET removal, DNase I and restoring efferocytosis are candidate therapeutic targets.

The current standard therapy for AAV, the combination of glucocorticoids with either cyclophosphamide or rituximab, could improve the survival rate (44, 45), but is problematically relevant to severe adverse events (46); thus, novel therapeutic strategies are needed to minimize side effects and improve the quality of life of patients with AAV. Based on the pathogenesis of AAV described in the previous section, therapeutic candidates for targeting neutrophils can be categorized as follows: neutrophil priming, intracellular signaling, and NETs processing (**Figure 1**).

### 4.1 Targeting neutrophils priming

#### 4.1.1 C5a receptor antagonist

The complement alternative pathway is involved in pathogenesis of AAV. In an MPO-ANCA transfer mouse model, C4-deficient mice develop vasculitis after injection of anti-MPO IgG, whereas C5- and factor B-deficient mice are resistant to the development of vasculitis (47). Interestingly, deficiency of C6, a component of membrane attack complex (MAC), has no effect on development of vasculitis, and C5a receptor deficiency and treatment with a C5a receptor antagonist ameliorates MPO-ANCA-induced vasculitis (48). C5a functions as a mediator of innate immunity, including activation of immune cells, particularly neutrophils. A high dose of C5a directly induce NETs formation, whereas a low dose primes neutrophils for ANCA-induced NETs formation (49–51). Avacopan, a C5a receptor antagonist, is now administered in clinical practice. The efficacy and safety of avacopan have been reported in a phase 3 randomized double-blind placebo-controlled clinical trial (ADVOCATE) (52). In this study, patients with AAV received oral avacopan at a dose of 30 mg twice daily or oral prednisone on a tapering schedule for 20 weeks in combination with cyclophosphamide or rituximab. The remission rates at weeks 26 and 52 in avacopan group were higher compared to those in prednisone group. Patients treated with avacopan showed significant improvement in kidney function at 26 and 52 weeks. The incidence of serious adverse events did not differ between the two groups, and the occurrence of glucocorticoid-induced toxic effects was lower in patients receiving avacopan. Although it should be noted that glucocorticoids were not completely eliminated in patients receiving avacopan, avacopan is a new treatment strategy which is able to replace glucocorticoids.

#### 4.1.2 Recombinant thrombomodulin

TM is a transmembrane glycoprotein that exists on the surface of vascular endothelium and exhibits anti-coagulant activity. During inflammation, TM plays an important role in suppressing vascular inflammation by regulating leukocyte recruitment and complement system (53, 54). Moreover, the addition of rTM protects organs against NET-mediated tissue injury (55, 56). In AAV, rTM suppresses ANCA-induced NETs by inhibiting the engagement of integrin on neutrophils (57, 58). Treatment with rTM improves vasculitis in active immune AAV model rats and spontaneous AAV model mice by reducing NET formation (58). rTM has been used in patients with disseminated intravascular coagulation (DIC) in Japan; therefore, further research is needed to confirm the efficacy of rTM in the pathogenesis of AAV.

#### 4.1.3 Semaphorin 4D

SEMA4D is a transmembrane protein which has functional roles in the immune system through the interactions with plexin B1 and B2 (59). Surface SEMA4D on neutrophils interacts with plexin B2 on endothelial cells and inhibits neutrophil activation by reducing ROS production by suppressing Rac1 activity. In patients with AAV, serum levels of soluble SEMA4D are elevated, and the expression of neutrophil surface SEMA4D is decreased owing to the proteolytic cleavage of membrane SEMA4D, which results in neutrophil activation and NETs formation (60). SEMA4D is cleaved by disintegrin and metalloproteinase domain-containing protein 17 (ADAM17), and serum levels of ADAM17 are also correlated with disease activity in PR3-AAV (61). These findings suggest that SEMA4D might be a therapeutic target, as well as a serological marker.

### 4.2 Targeting intracellular signaling after ANCA binding

#### 4.2.1 Syk inhibition

The engagement of the Fcγ receptor by the Fc region of ANCA in neutrophil induces phosphorylation of Syk and production of NOX-mediated ROS (25). Syk phosphorylation in neutrophils is increased in patients with active AAV, and is correlated with ANCA titers. Syk inhibition prevents the production of IL-8 and ROS by neutrophils *in vitro* (62). The treatment of fostamatinib, Syk inhibitor, is protective in AAV model rats induced by immunization with human MPO with the improvement of organ injury and and renal dysfunction (63). Syk is also required for the survival of B cells, which play a central role in AAV in that they produce pathogenic ANCA (64). Based on *in vitro* and *in vivo* studies, Syk inhibition is a promising approach to regulate intracellular signaling of neutrophil activation and affect acquired immunity in AAV.

#### 4.2.2 Cellular adhesion

Leukocyte-endothelial cell interactions mediated by Fcγ receptors and integrin β_2_ play an important role in vascular inflammation of AAV (65). Ex vivo studies using culture plates coated with human plasma fibronectin have shown that ANCA enhances β2 integrins in human neutrophils and induces NETs formation in accordance with the adhesion kinase pathway. The adhesion *via* the integrin is involved in actin polymerization in neutrophils. The downstream of adhesion pathway activates NOX and produces ROS, resulting in NETs formation (27). These indicate that substrate attachment is required for NET formation in response to ANCA, and that inhibition of the adhesion pathway could be a therapeutic target in AAV (27).

#### 4.2.3 ROS inhibition (MPO inhibitor and ROS inhibitor)

Inhibition of MPO with AZM198 decreases ROS production and NET formation in neutrophils stimulated with TNF-α and PR3-ANCA *in vitro* (66). Extracellular MPO released from neutrophils is frequently detected in kidney of patients with MPO-AAV and is associated with the presence of NETs, indicating that extracellular MPO may participate in renal injury and be a therapeutic target (67). AZM198 treatment in a nephrotoxic nephritis model, which causes crescentic glomerulonephritis, attenuates proteinuria, serum creatinine levels, and glomerular inflammation (66). NET formation is also reduced by treatment with the NOX inhibitor, DPI, under stimulation with TNF-α and anti-MPO antibody *in vitro* (27). ROS are key factors in NET formation, therefore further investigations focusing on ROS as therapeutic targets for AAV are required.

#### 4.2.4 PAD inhibitor

PAD enzymes convert arginine residues into citrulline. Five PAD enzymes are expressed in human, and PAD4 is mainly detected in white blood cells, including neutrophils (68, 69). Histone citrullination by PAD4 promotes NET formation through chromatin decondensation. Cl-amidine, a pan-PAD inhibitor, decrease NET formation in HL-60 cells by inhibiting histone citrullination (70), and PAD4-deficient neutrophils are unable to form NETs (12). *In vitro* and *in vivo* studies have demonstrated that ANCA induces PAD4-dependent NETs (5). In PMA plus PTU-induced mouse models with MPO-ANCA production, citrullination in the peritoneum and serum MPO-ANCA titer were reduced by treatment with Cl-amidine (71). Furthermore, a specific PAD4 inhibitor (GSK484) and pan PAD4 inhibitor ameliorated the murine model of AAV (72).

#### 4.2.5 Targeting necroptosis

Necroptosis is an inflammatory form of programmed necrosis characterized by cell membrane rupture and the release of DAMPs and is involved in the pathogenesis of various disease including acute organ injury and autoimmune diseases. Necroptosis is induced by cell death receptors, toll-like receptors, and other mediators, and the initiation of necroptosis requires the activation of RIPK3 and phosphorylation of MLKL (26). A recent study has shown the involvement of RIPK1-RIPK3-MLKL signaling pathway in ANCA-induced NET formation (4). RIPK1 inhibitor Nec-1s and MLKL deficiency reduce NET formation in response to anti-MPO IgG, and RIPK3- or MLKL-deficient mice are protected from necrotizing crescentic glomerulonephritis in a passive anti-MPO antibody transfer AAV model. Currently, clinical trials of RIPK1 inhibitors in patients with inflammatory diseases such as psoriasis, rheumatoid arthritis, and ulcerative colitis have been carried out (73). With the further development of programmed neutrophil necrosis in AAV, necroptosis inhibitors are expected to be effective for the treatment of AAV.

#### 4.2.6 CypD inhibitors

CypD exists in the mitochondrial matrix, and is a key regulator of mitochondrial permeability transition pore (mPTP). Accumulation of ROS along with mitochondrial calcium overload initiates mPTP opening, the release of mitochondrial ROS and cytochrome c into the cytoplasm, resulting in cell death (74–76). mPTP regulated by CypD interacts with necroptosis (77), which participates in the pathogenesis of autoimmune diseases. Cyclosporine A, an inhibitor of CypD, inhibits IL-8-induced NETosis *in vitro* (78). The pharmacologic and genetic inhibition of CypD reduce ANCA-induced NET formation, and CypD inhibitor protects endothelium from NET toxicity *in vitro* (5). In passive anti-MPO antibody transfer and spontaneous AAV mouse models, genetic ablation of CypD improves necrotizing vasculitis *via* the mPTP-related necrosis. Cyclosporine A is widely used in daily clinical practice. The successful use of cyclosporine A has been reported in a small number of patients with AAV (79); thus, CypD inhibition is a promising drug candidate for AAV.

Impressively, key molecules in various programmed cell death processes, including PADI4, RIPK, and CypD, are involved in ANCA-mediated neutrophil activation and the pathogenesis of AAV. Among these, the targets that have the greatest impact on AAV will be addressed in future studies.

### 4.3 Targeting NETs processing

#### 4.3.1 DNase I

DNase I is mainly produced by the pancreas and is essential for digestion of NETs (32). Serum DNase I activity is significantly lower in patients with AAV than in healthy individuals, resulting in persistence of NETs (35). *In vivo*, the immunization of naïve mice with myeloid dendritic cells co-cultured with NETs induces ANCA production and autoimmune vasculitis, whereas the immunization with pretreatment of DNase I reduced ANCA production and subsequent vasculitis (2), indicating that DNA released from NET-forming neutrophils is needed to maintain the antigenicity for ANCA production. Additionally, DNase I treatment in a passive anti-MPO antibody transfer AAV model protected mice from necrotizing crescentic glomerulonephritis (4).

DNase I is a potential therapeutic agent for reducing the viscosity of cystic fibrosis sputum. Currently, to prolong the half-life of DNase I, conjugation with polyethylene glycol (PEG) (80) or DNase I-coated nanoparticles (81) has been developed, and these properties could be a therapeutic option for NETs-related diseases, including AAV. Meanwhile, plasma exchange (PE) with albumin replacement in the PEXIVAS trial did not improve clinical outcomes, including death and end-stage kidney disease in patients with severe AAV (82); however, the use of fresh frozen plasma (FFP) in PE might be effective as a replacement of DNase I.

#### 4.3.2 Restored efferocytosis of ANCA-mediated NETs

Although NETs are mainly digested by DNase I in serum, physiological amounts of DNase I are insufficient for NETs-degradation in some conditions where macrophage engulfment assists NETs clearance (33, 34). The process of dead cell removal by macrophages, termed efferocytosis, is regulated by the CD47-mediated “don’t eat me” signal (83). Our laboratory has shown that kidney biopsy samples from patients with AAV and ex vivo ANCA-induced NETs show increased expression of CD47 (36). *In vitro*, ANCA-induced NETs with enhanced CD47 expression escape efferocytosis, and the blockade of CD47 with anti-CD47 antibody restored the efferocytosis of ANCA-induced NETs. *In vivo*, anti-CD47 antibody treatment at onset ameliorated renal injury and reduced MPO-ANCA production in serum, with decrease of NETs in glomeruli in spontaneous AAV model mice. However, blocking CD47 at the peak of the disease worsens disease activity in mouse models of systemic lupus erythematosus (SLE) and experimental autoimmune encephalomyelitis (EAE) (84, 85). The discrepancy is likely due to the difference in the timing of treatment initiation. Further studies are needed to reveal the mechanisms of CD47-signaling pathway including immune activation *via* CD47 blockade in AAV.

## 5 Conclusions

In this review, we describe the current and future treatment strategies by focusing on the mechanism of NETs formation and clearance, which play a key role in the pathogenesis of AAV. Disease-specific therapeutic agents are necessary to prevent the adverse effects of current immunosuppressive therapies due to broad immune suppression. It is noteworthy that a C5a receptor antagonist, which inhibits neutrophil priming, has become a treatment option in daily practice. Although therapy for AAV has improved, there are still unmet medical needs regarding the presence of therapy-resistant patients, treatment-related complications, and high relapse rates. Further studies are needed to develop novel therapeutic strategies that involve a pathogenesis-based approach, including the regulation of NETs in AAV.

## Author contributions

SS-A: Writing – original draft, Writing – review & editing. DN: Writing – original draft, Writing – review & editing.
